# Comparative analysis of dioecious *Amaranthus* plastomes and phylogenomic implications within Amaranthaceae s.s.

**DOI:** 10.1186/s12862-023-02121-1

**Published:** 2023-05-06

**Authors:** Damilola A. Raiyemo, Patrick J. Tranel

**Affiliations:** Department of Crop Sciences, University of Illinois, Urbana, IL 61801 USA

**Keywords:** *Amaranthus* species, Dioecious amaranths, Chloroplast genome, Phylogenetic analyses, Evolutionary distance

## Abstract

**Background:**

The genus *Amaranthus* L. consists of 70–80 species distributed across temperate and tropical regions of the world. Nine species are dioecious and native to North America; two of which are agronomically important weeds of row crops. The genus has been described as taxonomically challenging and relationships among species including the dioecious ones are poorly understood. In this study, we investigated the phylogenetic relationships among the dioecious amaranths and sought to gain insights into plastid tree incongruence. A total of 19 *Amaranthus* species’ complete plastomes were analyzed. Among these, seven dioecious *Amaranthus* plastomes were newly sequenced and assembled, an additional two were assembled from previously published short reads sequences and 10 other plastomes were obtained from a public repository (GenBank).

**Results:**

Comparative analysis of the dioecious *Amaranthus* species’ plastomes revealed sizes ranged from 150,011 to 150,735 bp and consisted of 112 unique genes (78 protein-coding genes, 30 transfer RNAs and 4 ribosomal RNAs). Maximum likelihood trees, Bayesian inference trees and splits graphs support the monophyly of subgenera *Acnida* (7 dioecious species) and *Amaranthus*; however, the relationship of *A. australis* and *A. cannabinus* to the other dioecious species in *Acnida* could not be established, as it appears a chloroplast capture occurred from the lineage leading to the *Acnida* + *Amaranthus* clades. Our results also revealed intraplastome conflict at some tree branches that were in some cases alleviated with the use of whole chloroplast genome alignment, indicating non-coding regions contribute valuable phylogenetic signals toward shallow relationship resolution. Furthermore, we report a very low evolutionary distance between *A. palmeri* and *A. watsonii*, indicating that these two species are more genetically related than previously reported.

**Conclusions:**

Our study provides valuable plastome resources as well as a framework for further evolutionary analyses of the entire *Amaranthus* genus as more species are sequenced.

**Supplementary Information:**

The online version contains supplementary material available at 10.1186/s12862-023-02121-1.

## Introduction

The genus *Amaranthus* L. consists of 70–80 species dispersed across the temperate and tropical regions of the world [1]. The genus has been described as taxonomically challenging and species identification can be difficult due to small or inconspicuous reproductive organs [2–4]. Accurate identification of species in the genus thus requires the use of habit, leaf size and shape, fruit type, bracts, bracteoles, and sepals of pistillate flowers. Species in the genus are characterized by their alternate distal leaves and unisexual flowers, which is distinct from closely related genera in the Amaranthaceae family with distal opposite leaves and bisexual flowers [4]. The genus is divided into three subgenera, *Amaranthus* subgenus *Amaranthus*, *Amaranthus* subgenus *Albersia* (Kunth) Gren. & Godr. and *Amaranthus* subgenus *Acnida* (L.) Aellen ex K.R. Robertson [5].

The subgenus *Acnida* is made up of nine dioecious species that are native to North America and is further classified into three sections, *Acnida* sect. *Acnida* (L.) Mosyakin & K.R. Robertson [comprised of *A. australis* (A. Gray) J.D. Sauer, *A. cannabinus* (L.) J.D. Sauer, *A. floridanus* (S. Watson) J.D. Sauer, *A. tuberculatus* (Moq.) J.D. Sauer], *Acnida* sect. *Acanthochiton* (Torr.) Mosyakin & K.R. Robertson [comprised of *A. acanthochiton* J.D. Sauer] and *Acnida* sect. *Saueranthus* Mosyakin & K.R. Robertson [comprised of *A. arenicola* I.M. Johnson, *A. greggii* S. Watson, *A. watsonii* Standley, and *A. palmeri* S. Watson] [5–9]. The infrageneric classification above was based on combinations of morphological characteristics: dehiscent or indehiscent fruits, presence/absence of foliaceous bracts, presence/absence of tepals of pistillate flowers, shape of the tepals and whether they are well developed or not [5–7].

Several species within the *Amaranthus* genus are economically important in that they offer nutritional benefits and are either grown for their grains (e.g., *A. hypochondriacus* L., *A. cruentus* L. and *A. caudatus* L.) or as leafy vegetables in parts of Asia and Africa (e.g., *A. tricolor* L., *A. blitum* L. and *A. dubius* L.) [10–13]. However, twenty species are widespread as weeds of crop lands and non-agrarian areas around the world, with *A. tuberculatus* and *A. palmeri* being particularly troublesome due to their rapid adaptability to changing climatic conditions, management strategies and herbicide management [11, 14, 15]. Investigation of species’ relationships within the genus could enable better comprehension of trait evolution (e.g., weediness).

Previous studies investigating the relationships among the amaranths have utilized either plastid DNA markers (e.g., *matK*, *trnL*), nuclear ribosomal internal transcribed spacer (ITS), low-copy nuclear genes (e.g., *Waxy*, *A36*), nuclear markers (e.g., *ALS*, AFLP), biallelic single nucleotide polymorphisms or chloroplast genomes [16–21]. Waselkov et al. [20] in their phylogenetic studies reported partial support for the infrageneric classification of Mosyakin and Robertson [5], with grouping of some species corresponding to the three subgenera. It was however noted that the infrageneric taxa may not reflect the evolutionary history of species in the genus [20, 22]. Moreover, many of the previous phylogenetic studies have either sequenced and assembled chloroplast genomes as genomic resource and sampled very few dioecious species or used few markers for tree construction. Neither strategy has offered convincing support for the relationships among the dioecious *Amaranthus* species.

Chloroplast genomes provide an advantage in inferring evolutionary relationships among species because they are highly conserved with stable gene content, gene order and overall lower substitution rates relative to nuclear genomes [23, 24]. They have a typical quadripartite structure consisting of a large single copy region (LSC), a small single copy region (SSC) and a pair of inverted repeats (IRs) with small sizes ranging from 115 to 165 Kb for most photosynthetic organisms [25–27]. Although methods including plastid DNA enrichments and bacterial artificial chromosome (BAC) were earlier proposed to obtain chloroplast genomes from plants [26], advances in genome sequencing, bioinformatics and phylogenomic methods have simplified the acquisition of chloroplast genomes using next-generation sequencing as well as their subsequent analysis [28–30]. Complete chloroplast genomes thus possess more parsimony-informative sites and, in many cases, provide better resolution in deciphering species relationships than do a few molecular markers [31–33].

There are about 23 *Amaranthus* species’ plastomes available in public repositories; some with incomplete annotations and others remain unverified after author’s submission [NCBI GenBank database [34], accessed on July 7, 2022]. The low number of available chloroplast sequences for species in the *Amaranthus* genus is thus insufficient. In this study, we report the complete chloroplast sequence data for the nine dioecious species of the *Amaranthus* genus. The objectives of this study are to (1) investigate the structural organization of plastomes of dioecious *Amaranthus* species, (2) identify divergence hotspots that could be useful in species delimitation or development of barcoding markers and (3) provide a comprehensive plastid-based phylogenetic resource for comparison with tree topologies that are derived from nuclear genomes. In addition to seven newly sequenced and assembled plastomes of dioecious *Amaranthus* species, we further assembled plastomes from previously reported short reads of species in the family Amaranthaceae s.s. for comparative analyses.

## Results

### Characteristics of the dioecious *Amaranthus* chloroplast

**Fig. 1:**
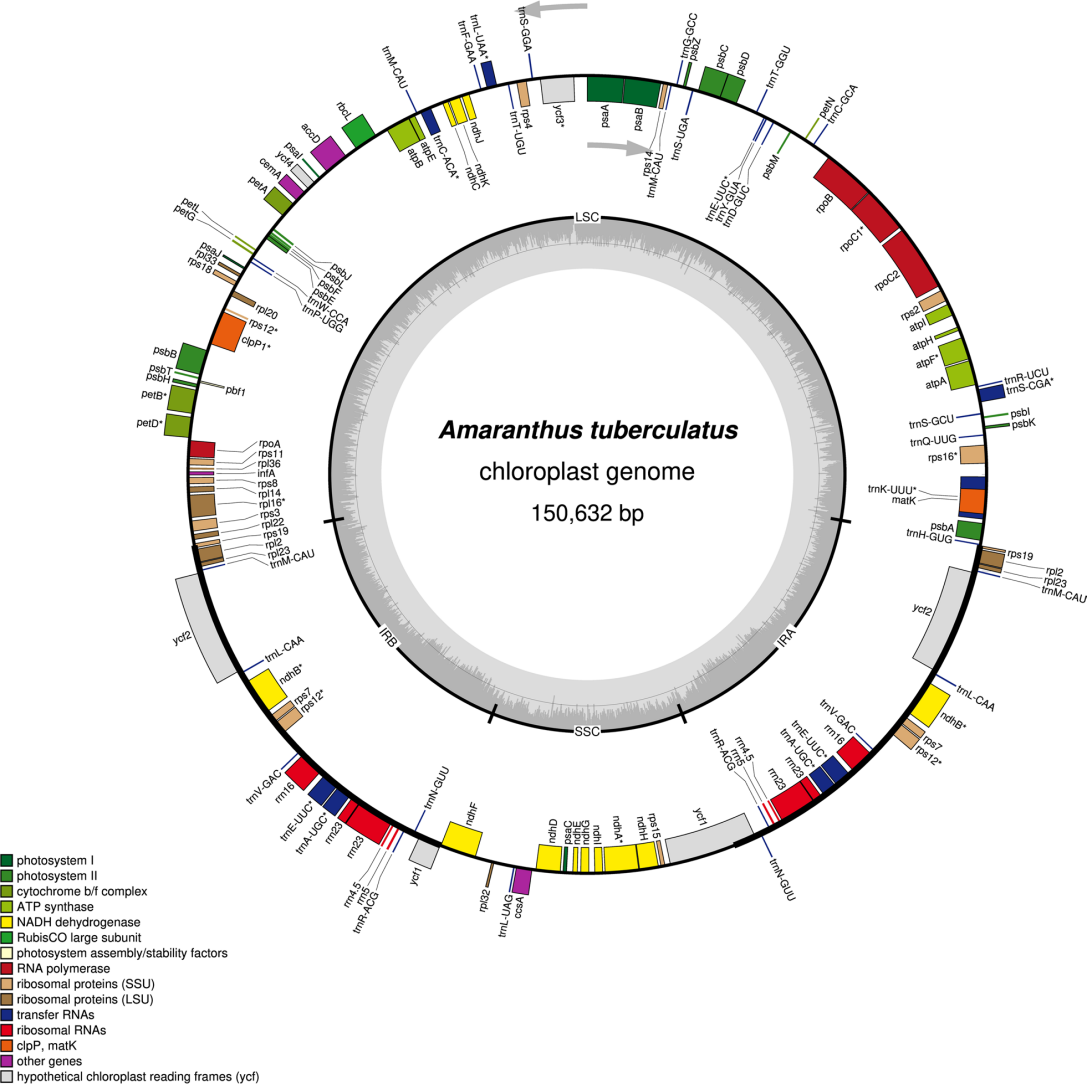
Annotated chloroplast gene map of *Amaranthus tuberculatus*. Genes depicted on the inside of the circle are transcribed clockwise while genes shown on the outside of the circle are transcribed counterclockwise. Genes with asterisk have introns. The dark grey area within the circle represents the GC content across the chloroplast genome

Raw reads data from which seven dioecious *Amaranthus* chloroplast genomes were assembled are available under the NCBI Sequence Read Archive (SRA) project number PRJNA836903 while information on the other two dioecious species is provided in the supplementary file (Additional file 1: Table S2). The assembled chloroplast genomes of the nine dioecious *Amaranthus* species ranged from 150,011 bp (*A. australis*) to 150,735 bp (*A. greggii*). The genomes have a typical quadripartite structure consisting of a large single copy (LSC) region (83,244–83,986 bp), and a small single copy (SSC) region (18,026–18,088 bp), separated by two inverted repeat (IR) regions (24,346–24,352 bp) (Fig. 1, Table 1). The average GC content for the nine genomes ranged from 36.56 (*A. cannabinus*) to 36.62 (*A. australis*) (Table 1). The genomes contained 133 genes including 88 protein-coding genes, 37 tRNA genes and 8 rRNA genes. The LSC region contained 83 genes out of which 61 were protein-coding and 22 were tRNAs, while the SSC region contained 11 protein-coding genes and 1 tRNA. The IR region (IRb) contained 17 genes (6 protein-coding, 7 tRNAs and 4 rRNAs) and a *ycf1* fragment while IRa also had the 17 genes present in IRb and an *rps19* fragment. The partial fragments of both *ycf1* and *rps19* in the *Amaranthus* chloroplast genomes are consistent with previous reports for chloroplast genomes that have suggested the pseudogenization of both genes [35–37]. There were 17 distinct genes (*ndhB*, *petB*, *petD*, *atpF*, *clpP1*, *ndhA*, *rpl16*, *rpoC1*, *rps12*, *rps16*, *pafI*, *trnG*^*UCC*^, *trnI*^*GAU*^, *trnL*^*UAA*^, *trnA*^*UGC*^, *trnK*^*UUU*^, *trnV*^*UAC*^) with introns, in which 3 (*rps12*, *clpP1* and *ycf3*) had two introns. The gene *trnK*^*UUU*^ had the longest intron at 2,586 bp. Overall, 78 protein-coding genes, 30 tRNA genes and 4 rRNA genes, making a total of 112 genes, represent the unique genes found in the chloroplast genomes of dioecious *Amaranthus* species (Table 1). Although Geseq annotated the gene *rpl23* in the genomes, Chloe did not annotate this gene. Previous studies have reported the pseudogenization of *rpl23* in the order Caryophyllales and several angiosperm taxa [38, 39]. We therefore did not consider it further in subsequent analysis.

**Table 1:** Chloroplast genome features of nine dioecious *Amaranthus* species

Species	Length (bp)	Coverage depth (x)	LSC (bp)	SSC (bp)	IR (bp)	GC (%)	Number of unique genes			
							Protein-coding	tRNA	rRNA	Total
*A. acanthochiton*	150,653	522.6	83,927	18,034	24,346	36.59	78	30	4	112
*A. arenicola*	150,655	511.4	83,926	18,037	24,346	36.61	78	30	4	112
*A. australis*	150,011	615.2	83,244	18,065	24,351	36.62	78	30	4	112
*A. cannabinus*	150,677	774.8	83,888	18,085	24,352	36.56	78	30	4	112
*A. floridanus*	150,670	514.0	83,935	18,043	24,346	36.60	78	30	4	112
*A. tuberculatus*	150,632	740.1	83,901	18,039	24,346	36.61	78	30	4	112
*A. greggii*	150,735	519.1	83,955	18,088	24,346	36.58	78	30	4	112
*A. watsonii*	150,706	520.6	83,986	18,026	24,347	36.61	78	30	4	112
*A. palmeri*	150,708	484.5	83,988	18,026	24,347	36.60	78	30	4	112

*LSC* large single copy, *SSC* small single copy, *IR* inverted repeat

### Simple sequence repeats (SSRs), repetitive sequences and codon usage bias patterns

Simple sequence repeats in the chloroplast genomes of the nine dioecious *Amaranthus* species ranged from 31 (*A. acanthochiton*) to 37 (*A. cannabinus*), of which the mononucleotides (10–17) and tetranucleotides (10–14) repeats were most abundant. All nine species had one hexanucleotide SSR while only *A. cannabinus* had one pentanucleotide repeat (Table 2). Composition of repetitive sequence types across the species ranged from 36 in four species (*A. acanthochiton*, *A. cannabinus*, *A. watsonii* and *A. palmeri*) to 39 in *A. greggii*. Forward and palindromic repeats across the species ranged from 14–16 and 21–23, respectively. One reverse repeat was identified in all species except *A. acanthochiton*, *A. australis* and *A. cannabinus*, which had none. No complementary repeat was detected in any of the nine species at the threshold used to find the repeats (Table 3).

**Table 2:** Simple sequence repeats (SSRs) in the nine dioecious *Amaranthus* chloroplast genomes

Species	Mono	Di	Tri	Tetra	Penta	Hexa	Compound	Total
*A. acanthochiton*	12	2	4	11	0	1	1	31
*A. arenicola*	13	2	4	10	0	1	2	32
*A. australis*	10	2	5	12	0	1	3	33
*A. cannabinus*	14	2	4	14	1	1	1	37
*A. floridanus*	17	2	4	10	0	1	2	36
*A. tuberculatus*	15	2	4	10	0	1	2	34
*A. greggii*	14	1	4	11	0	1	1	32
*A. watsonii*	12	3	4	11	0	1	1	32
*A. palmeri*	12	3	4	11	0	1	1	32

**Table 3:** Number of repetitive sequence types in nine dioecious *Amaranthus* chloroplast genomes

Species	Forward	Palindrome	Reverse	Complement	Total
*A. acanthochiton*	14	22	0	0	36
*A. arenicola*	14	22	1	0	37
*A. australis*	15	23	0	0	38
*A. cannabinus*	15	21	0	0	36
*A. floridanus*	14	22	1	0	37
*A. tuberculatus*	14	23	1	0	38
*A. greggii*	16	22	1	0	39
*A. watsonii*	14	21	1	0	36
*A. palmeri*	14	21	1	0	36

Codon usage frequency is believed to differ across genomes or among genes, and codons that are optimal are important for efficient and accurate translation [40–42]. The codon usage and relative synonymous codon usage (RSCU) for the *A. tuberculatus* chloroplast genome was calculated based on 78 protein-coding sequences in the genome (61 within the LSC, 6 within IR and 11 within the SSC regions). The 78 protein-coding genes were encoded by 21,260 codons, excluding stop codons (Additional file 2: Table S5). Codons with the third-position nucleotide of A or T were used more often than codons ending with G or C. The most common amino acid codon in the* A. tuberculatus* cp genome was leucine at 2,233 codons (10.5%), while the least frequent was cysteine at 665 codons (3.12%) (Additional file 2: Table S5).

### Comparative analysis of dioecious *Amaranthus* chloroplast genome structure

**Fig. 2:**
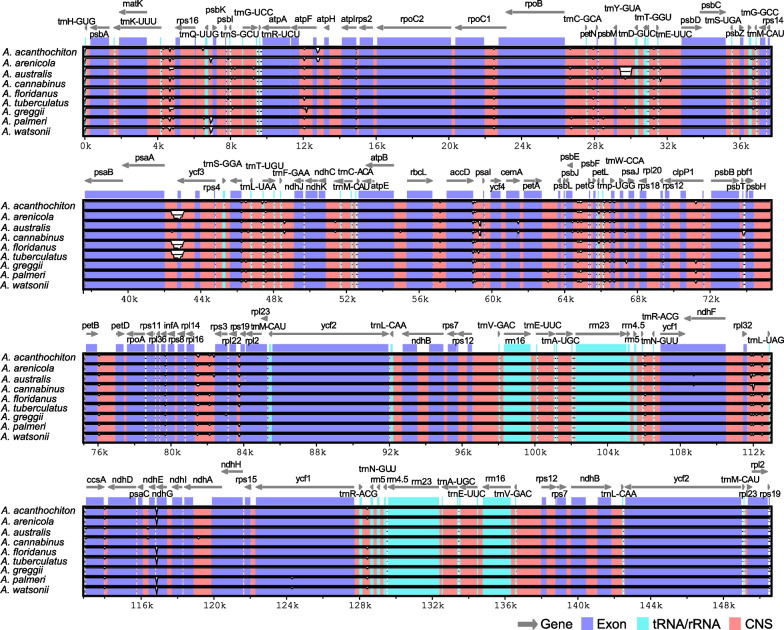
Sequence alignment of complete chloroplast genomes of nine dioecious *Amaranthus* species to the *A. hypochondriacu*s chloroplast genome (KX279888) using mVISTA. The y-axis within each species bar corresponds to percentage sequence identity (50–100%). The grey arrows indicate annotated genes within the genomes and their transcriptional direction. Genomic regions are color-coded as protein-coding (exon), transfer or ribosomal RNA (tRNA/rRNA), and conserved non-coding sequences (CNS)

**Fig. 3:**
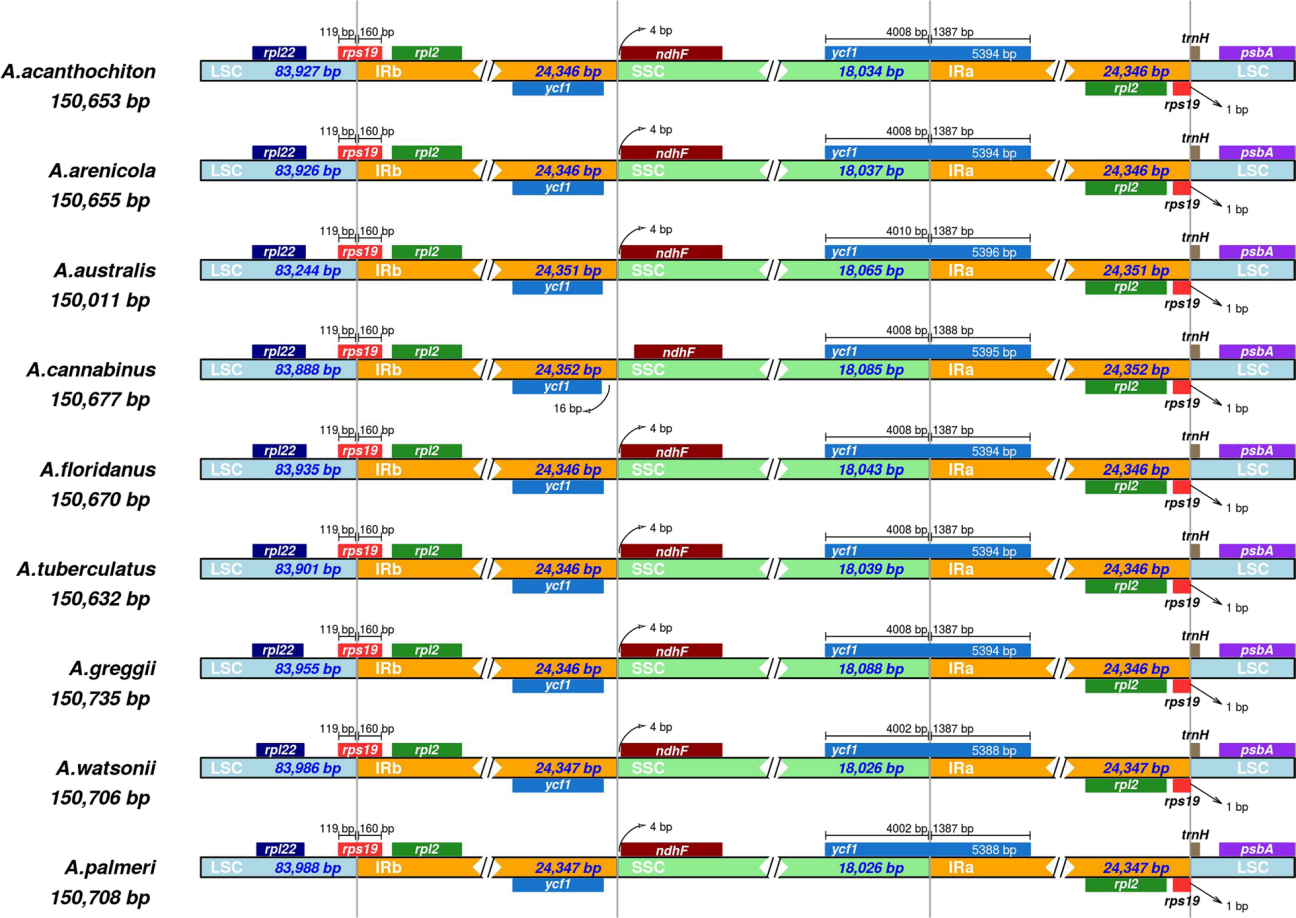
Comparison of large single copy (LSC), small single copy (SSC) and inverted repeats (IR) border regions among the nine dioecious *Amaranthus* chloroplast genomes. Genes preceded by the Greek letter psi (ψ) represent possible pseudogenes

**Fig. 4:**
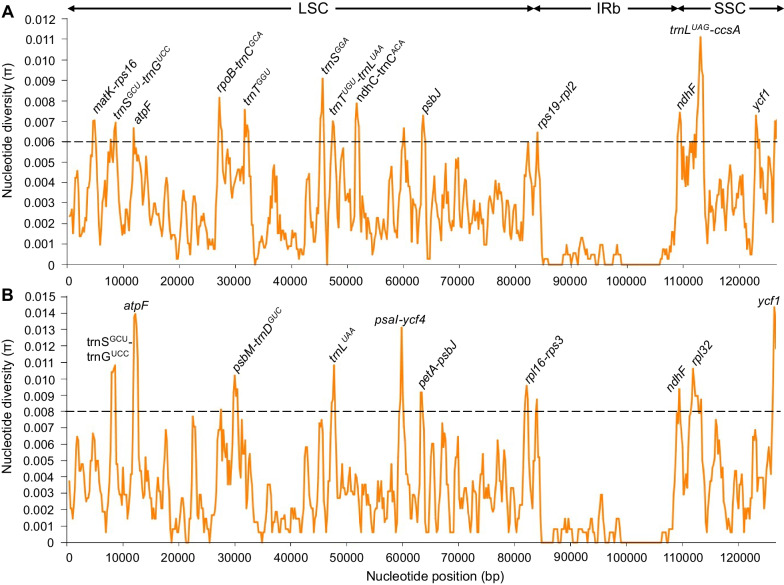
Sliding window analysis of nucleotide diversity within *Amaranthus* plastomes. **A** Comparison among nine dioecious species and **B** comparison among four weedy species: *A. tuberculatus*, *A. palmeri*, *A. hybridus* and *A. retroflexus* (GenBank Accession number MW646089). Window length: 800 bp; step size: 200 bp

Pairwise comparison of sequence divergence across the nine dioecious *Amaranthus* species and the reference *A. hypochondriacus* chloroplast genome using mVISTA revealed highly conserved coding regions while the non-coding regions were more divergent (Fig. 2). Although the intergenic region *psaA*-*ycf3* appears to be more conserved across six species, it appears to be less conserved across *A. arenicola*, *A. floridanus* and *A. tuberculatus*. The intergenic region *psbM*-*trnD*^*GUC*^ also showed a high divergence in *A. australis*. Other intergenic regions, such as *rpl32*-*trnL*^*UAG*^, *trnK*^*UUU*^-*rps16*, *trnS*^*GCU*^-*trnG*^*UCC*^, and *ndhE*-*ndhG*, also exhibited variations relative to the reference. These intergenic spacer regions have been reported to be variable in other plant species and hold valuable phylogenetic signals for resolving species’ relationships [43–47]. Analysis of the LSC/IRb/SSC/IRa boundaries showed that *rps19* is located at the boundary of LSC/IRb with 119 bp of its length within the LSC region and 160 bp of its length within IRb region, while *ycf1* is located at the SSC/IRa boundary with 4008 bp of its length within the SSC region and 1387 bp of its length within the IRa region (Fig. 3). Contraction and expansion of IR regions contribute to size variation and rearrangement of the LSC/IRb/SSC/IRa boundaries in angiosperms [48]. However, there were no differences between the LSC/IRb, IRb/SSC, and SSC/IRa boundaries across the nine dioecious *Amaranthus* species in our study (Fig. 3). Thirteen mutational hotspots (9 in LSC, 3 in SSC and 1 in IR regions) exhibited nucleotide diversity, π, greater than 0.006 when comparing the nine dioecious species (Fig. 4A) while ten hotspots (7 in LSC and 3 in IR regions) exhibited π greater than 0.008 when comparing four weedy *Amaranthus* species (Fig. 4B). Across the 19 *Amaranthus* species with available plastome sequences, twelve hotspots exhibited π greater than 0.008 (Additional file 3: Fig. S1). The overall low nucleotide variability among the *Amaranthus* species indicates high level of sequence conservation.

### Phylogenetic analysis

**Fig. 5:**
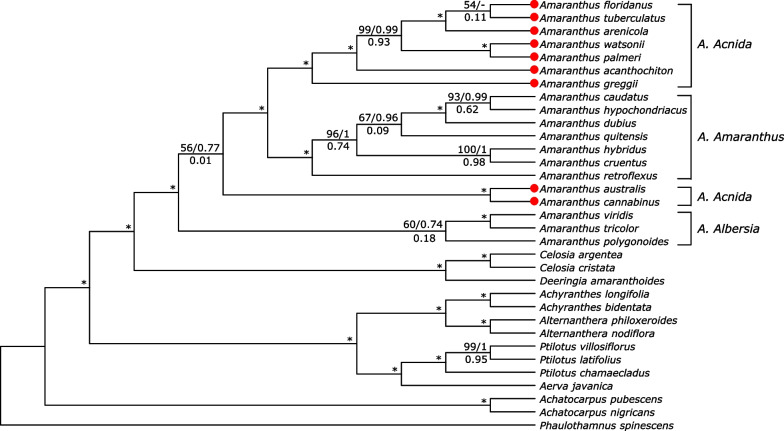
Phylogenetic tree of *Amaranthus* species and other species in Amaranthaceae s.s. based on 78 plastid protein-coding genes. Numbers above branches represent RAxML maximum likelihood bootstrap support (BS) and Bayesian posterior probability (PP) values, while values below branches represent Internode Certainty All (ICA) values. Asterisks indicate full support (BS = 100, PP = 1, ICA = 1.00). Terminal tips in red represent newly assembled plastid genomes in this study

There were 58,259 conserved sites, 9073 variable sites and 7203 parsimony-informative sites in a total of 67,333 alignments for the concatenated 78 protein-coding genes. Maximum likelihood and Bayesian inference phylogeny revealed high support for many branches on the tree, including the additional taxa belonging to 8 other genera in Amaranthaceae s.s., with bootstrap support values close to 100 and posterior probabilities close to 1. We recovered the monophyly of the subgenera *Acnida* (dioecious species) and *Amaranthus* (monoecious species), which corresponds to previously reported classification based on morphology (Fig. 5) [2, 5, 20]. Seven dioecious species (*A. tuberculatus*, *A. floridanus*, *A. arenicola*, *A. watsonii*, *A. palmeri*, *A. acanthochiton*, and *A. greggii*) within the subgenus *Acnida* formed a monophyletic group with full support (BS = 100, PP = 1, ICA = 1.00). Within this clade, the relationship of *A. tuberculatus* to *A. floridanus* was less supported (BS = 54, ICA = 0.11) although both species were sister to *A. arenicola*. Two other dioecious species, *A. australi*s and *A. cannabinus*, formed a clade but were less supported in their relationship with the *Acnida* + *Amaranthus* clades (BS = 56, PP = 0.77).

**Fig. 6:**
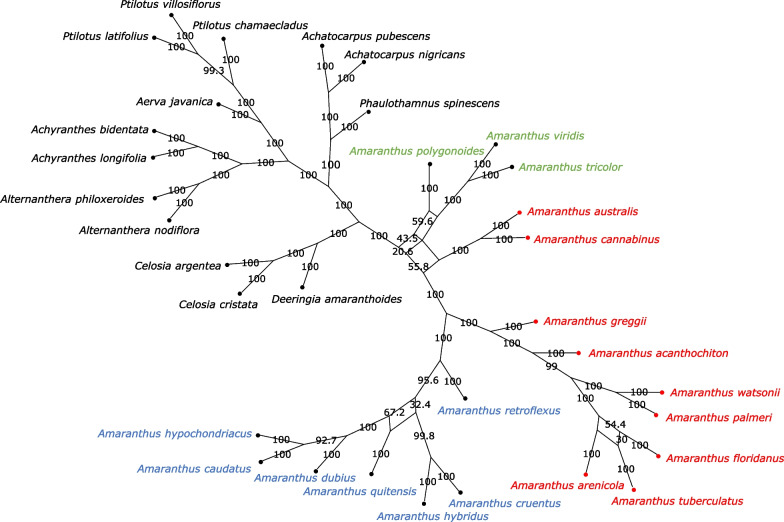
Bootstrap consensus network inferred from the maximum likelihood tree analysis for *Amaranthus* species and other species in Amaranthaceae s.s. based on 78 plastid protein-coding genes. Filtering threshold was 0.2, i.e., display splits or taxon bipartitions that occurred in at least 20% of the bootstrap replicates. Numbers on edges of the splits network are bootstrap support values. Species in red denotes subgenus *Acnida* while terminal tips in red are species with chloroplast genomes assembled in this study. Species in blue represents the subgenus *Amaranthus* while species in green represent subgenus *Albersia*

**Fig. 7:**
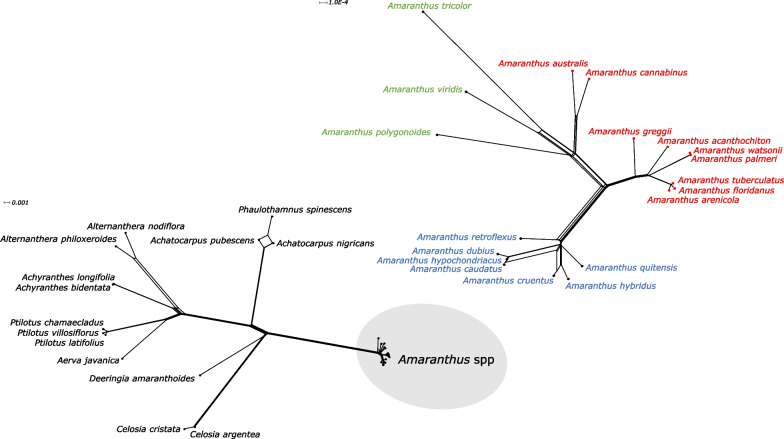
NeighborNet splits graph of *Amaranthus* species and other species in Amaranthaceae s.s. based on 78 plastid protein-coding genes. Split graph of *Amaranthus* spp. in the gray circle is enlarged in the top right. Species in red denotes subgenus *Acnida* while terminal tips in red are species with chloroplast genomes assembled in this study. Species in blue represents the subgenus *Amaranthus* while species in green represent subgenus *Albersia*. Scale bars (substitutions per site) are presented at the top-left corner of the graphs

The low ICA scores, 0.01 and 0.09, for the branch leading to a common ancestor between *A. australis*, *A. cannabinus*, and *Acnida* + *Amaranthus* clades, and the branch leading to *A. quitensis*, *A. dubius*, *A. hypochondriacus* and *A. caudatus*, respectively, indicates that the two most prevalent conflicting bipartitions have almost similar or at least close frequency of support (Fig. 5). Bootstrap consensus network also revealed that while 55.8% support the first bipartition leading to a common ancestor between *A. australis*, *A. cannabinus* and *Acnida* + *Amaranthus* clades, 43.5% support the second bipartition or branch leading to *A. australis*, *A. cannabinus* and species in the *Albersia* subgenus (Fig. 6). Similarly, 54.4% support the first bipartition or branch leading to *A. floridanus* and *A. tuberculatus* while 30% support the second bipartition or branch leading to *A. arenicola* and *A. tuberculatus* (Fig. 6). Although NeighborNet fit for the 78 CDS was 99.185%, indicating that the data is tree-like or bifurcating, the incongruence among the tree described above was further confirmed in the splits graph, thus corroborating the bootstrap consensus network (Fig. 7).

Quartet concordance (QC), quartet differential (QD) and quartet informativeness (QI) (collectively referred to as Quartet internodal score) indicate strong or perfect support for many of the tree branches i.e., 1/-/1 (Additional file 4: Fig. S2); however, the branch leading to *A. floridanus* and *A. tuberculatus* had a low QI score (0.067), similar to the branch leading to the common ancestor between *A. floridanus*, *A. tuberculatus*, *A. arenicola*, *A. watsonii* and *A. palmeri* (QI = 0.18), an indication of low information for the branches. The relationship between some species in the subgenus *Amaranthus* also appears to be weak with QC scores ranging from 0.068 to 0.51, QD scores from 0 to 0.52, and QI scores from 0.36 to 0.97. A low score for the three measures reflects a weak consensus relationship among species, possibility of competing alternative history or presence of a supported secondary evolutionary history, perhaps due to introgressive gene flow, and in some cases low information for branches. The relationship between *A. australis*, *A. cannabinus* and other dioecious *Amaranthus* spp. based on ICA was not clear as evidenced in the counter-support for the branch leading to a common ancestor between the two species and the *Acnida* + *Amaranthus* clades (QC = − 0.43, QD = 0.045). Overall, there was full support along the backbone relating the *Acnida* clade (seven dioecious species) and the *Amaranthus* clade (Additional file 4: Figure S2). Quartet Fidelity (QF) scores for the 33 taxa ranged from 0.6 to 0.94, indicating that many of the taxa sampled in this study were not misplaced (a term sometimes referred to as “rogue” taxa) (Additional file 4: Fig. S2).

Approximately unbiased (AU) test to determine if there is significant difference between trees with or without partitioning revealed both approaches were not significantly different (*p* > 0.5), therefore, results of the partitioned tree in IQTREE are presented and discussed. The topology and support for the tree generated in IQTREE adopting an optimal model was similar to the tree from RAxML (Additional file 4: Fig. S3). Although many branches had high support, the gene concordance factor (gCF) and site concordance factor (sCF) values corroborate the discordance or conflicts among branches earlier reported (Additional file 4: Fig. S3). For instance, the branch leading to *A. floridanus*, *A. tuberculatus* and *A. arenicola* had a 100% BS; however, only 19% of the genes and 98% of the sites are concordant with the focal branch. Also, the gCF calculated in IQTREE corresponds to the conflicting/concordant bipartitions among gene trees obtained in Phyparts (e.g., for a gCF value of 15.4% for the branch leading to *A. floridanus* and *A. tuberculatus*, only 12 genes out of 78 support that branch) (Additional file 4: Fig. S4). Interestingly, the level of discordance in gene trees is less pronounced for the other species of Amaranthaceae s.s. included in the tree as could be observed in the proportion of gene trees that supports their branches, further indicating that complex conflicts exist within the *Amaranthus* genus. Considering the “backbone” of *Amaranthus* using the 19 species, 71 genes support the backbone phylogeny or species tree while only 7 genes were discordant (Additional file 4: Fig. S4), similar to Morales-Briones et al. [49] where 62 genes were in concordance with the species tree for the *Amaranthus* genus while only 6 were discordant (see Additional Figure S5 in Morales-Briones et al.).

The test of topology based on approximately unbiased (AU) test to determine if an a priori constraint tree where all dioecious species are placed together would be better than an unconstraint tree revealed that the constraint tree is significantly different from the unconstraint one (*p* = 6e−07). The result of the AU test is also congruent with an initial log-likelihood test (Shimodaira-Hasegawa test) reported in RAxML, with the constraint tree indicted as significantly worse than the unconstraint tree (RAxML does not output *p*-values for log-likelihood tests). The topology test thus suggests that the two species *A. australis* and *A. cannabinus* are less closely related to the other dioecious amaranths based on their chloroplast genomes.

**Fig. 8:**
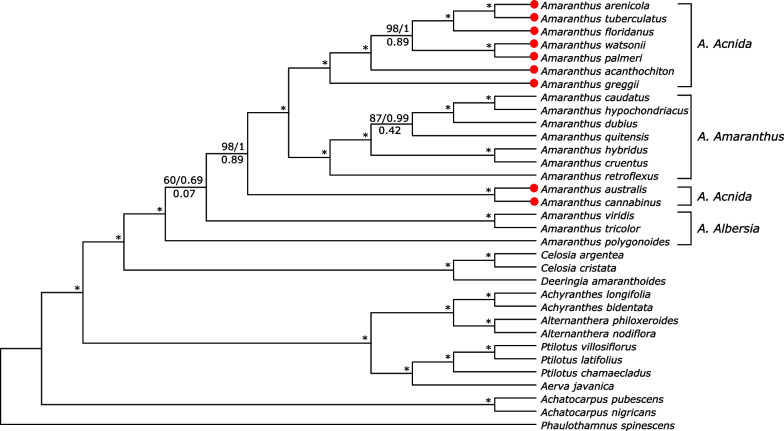
Phylogenetic tree of *Amaranthus* species and other species in Amaranthaceae s.s. based on whole chloroplast genomes. Numbers above branches represent IQ-TREE maximum likelihood ultrafast bootstrap support (UFBoot) and Bayesian posterior probability (PP) values, while values below branches represent RAxML Internode Certainty All (ICA) values. Asterisks indicate full support (BS = 100, PP = 1, ICA = 1.00). Terminal tips in red represent newly assembled plastid genomes in this study

For the plastome alignment excluding IRa, there were 103,019 conserved sites, 23,246 variable sites and 18,803 parsimony-informative sites in a total of 126,265 columns. The topology of the tree using 78 plastid protein-coding genes and whole plastome sequences were very similar, except the sister relationship between *A. arenicola* and *A. tuberculatus* was now established and had full support (BS = 100, PP = 1, ICA = 1.00). *Amaranthus australi*s and *A. cannabinus* once again did not cluster with the other dioecious species; however, the support for their relationship with the *Acnida* + *Amaranthus* clades increased (BS = 98, PP = 1, ICA = 0.89). Support values for other nodes also increased (Fig. 8). There was also no difference in topology and bootstrap support between IQTREE (TVM + F + R2 model) and RAxML (GTRGAMMA model) trees, except the node that had 60% bootstrap support in IQTREE had 49% bootstrap support in RAxML, therefore results from IQTREE are presented (see Additional file 4: Fig. S5 Bootstrap consensus network for RAxMLbootstrap support values). Bootstrap values measure the standard error of the inferred tree mean from a full dataset in which the standard error decreases with more samples or loci [50]; therefore, bootstrap support values are expected to be higher for the whole plastome alignment as opposed to the set of 78 protein-coding genes. Bootstrap consensus network and NeighborNet splits graph (fit = 99.661%) also showed a highly supported bipartition for *A. arenicola* + *A. tuberculatus*, and *A. australi*s + *A. cannabinus* lineages. However, 48.8% support the first bipartition or branch leading to *A. polygonoides* and the other species in Amaranthaceae s.s. while 32.6% support the second bipartition or branch leading to *A. viridis*, *A. tricolor* and other species in Amaranthaceae s.s. (Additional file 4: Figs. S5, S6). The Quartet internodal scores (QC/QD/QI) for the cp genome alignment for most branches, including the other species of Amaranthaceae s.s., was 0/0/1, respectively while taxon QF score ranged from 0.03 to 0.3 (data not shown). These scores differ considerably from the Quartet internodal scores obtained with the 78 protein-coding sequences, thus reflecting a very complex conflict that could not be resolved from modeling the evolution of the species while assuming the concatenated plastid supermatrix as a “single-gene”.

### Evolutionary distance between *A. palmeri* and *A. watsonii*

Adjusting the method for distance calculation by using *p*-distance, Maximum Composite Likelihood, LogDet or changing rates to Gamma or Gamma and a proportion of invariable sites, or changing the Gamma rate parameter to 8 had no noticeable effects on the distances calculated. Therefore, we report the uncorrected *p*-distances. The evolutionary distance between *A. palmeri* and *A. watsonii* based on cp genome (minus IRa) was 0.0000476, which is considerably low compared to the distances between *A. tuberculatus* and *A. arenicola* (0.000143), *A. tuberculatus* and *A. floridanus* (0.000254) and *A. arenicola* and *A. floridanus* (0.000254). *Amaranthus australis* and *A. cannabinus* have also been shown to be sister taxa, however, the distance between both species was higher (0.0021688). The internal transcribed spacer (ITS) and full nuclear ribosomal cistron (rDNA) regions were 5819 and 10,674 bp, respectively. Assembly size for the full rDNA ranged from 9894–11,582 bp (Additional file 1: Table S4). A BLAST search of 722 bp *A. tuberculatus* ITS (GenBank accession number MG685285) from Waselkov et al. [20] against our assembled *A. tuberculatus* nuclear rDNA revealed 96.8% similarity to a region in the assembly, suggesting that the assembly contained the complete ITS sequence region used in their study. Evolutionary distance between *A. palmeri* and *A. watsonii* and between *A. caudatus*, *A. cruentus* and *A. quitensis* based on the ITS region was 0.000000 (Additional file 5). The very low distance (0) between these species indicates the low informativeness of the ITS region in distinguishing between the species. Only 38 parsimony-informative sites were found in the ITS region across the 14 *Amaranthus* species with short reads available for rDNA assembly. When the full rDNA assembly (containing sequences from ETS and possibly IGS) was used for distance calculation, the distance between *A. palmeri* and *A. watsonii* was still low (0.000453) relative to the distances between *A. tuberculatus* and *A. arenicola* (0.003036), *A. tuberculatus* and *A. floridanus* (0.006462), and *A. arenicola* and *A. floridanus* (0.003645). The evolutionary distance between *A. hybridus* and *A. quitensis* was 0.016139, similar to the distance between *A. cruentus* and *A. quitensis* (0.016233) (Additional file 6).

## Discussion

### Dioecious *Amaranthus* species’ plastome features

We report the complete chloroplast genomes of nine dioecious *Amaranthus* species and their composition. The size of the cp genomes is consistent with the size of 150–151 kb reported for other *Amaranthus* species [21, 51]. Similarly, GC content, number of protein-coding genes, transfer RNAs, ribosomal RNAs and overall structure are highly conserved across the dioecious *Amaranthus* species. Our comparative analysis revealed regions that differed across the species e.g., *trnL*^*UAG*^-*ccsA*-*ndhD*, were highly divergent across the nineteen *Amaranthus* species and could be valuable in marker development or DNA barcoding. This region among others has been reported to be very variable across flowering plants [52, 53]. Moreover, the low nucleotide diversity (see Additional file 3: Fig. S1 for highest π value at 0.016) among *Amaranthus* species also suggests a high genetic similarity, which may impact phylogenetic signals. A similar pattern of low nucleotide variability was observed among species of *Aldama* (Asteraceae), where the most variable region had a π value between 0.02936 and 0.0305 [54]. Although chloroplast size variation in several species could be attributed to expansion and contraction of IR regions [55–57], the LSC/IRb/SSC/IRa boundaries, including their positions, were very conserved across the dioecious amaranths. Our analysis of microsatellites and repeats also revealed patterns consistent with previous studies of SSRs and repetitive sequences in the amaranths [21, 51]. The relative synonymous codon usage for dioecious amaranths is also similar to *A. hypochondriacus* and other plant cp genomes [51, 58].

### Phylogenetic incongruence among the dioecious amaranths

Of particular interest to us is the relationships among the dioecious amaranths, which have been elusive. Waselkov et al. [20] studied the phylogeny of the amaranths using six molecular markers and attributed observed cytonuclear tree discordance to incomplete lineage sorting (ILS) and chloroplast capture. Xu et al. [21], although they did not sample all dioecious amaranths, produced trees using complete chloroplast sequences but did not detect tree topology incongruence. Nontree-like signals in a phylogenetic tree could be due to either statistical reasons (incorrect model specification, sequence errors or short alignments) or biological factors such as hybridization, incomplete lineage sorting, ancestral gene flow or low mutation rate [59]. We therefore evaluated if factors including poor loci resolution contributes to gene tree incongruence and if the use of more markers could provide better phylogenetic resolution.

Using a series of complementary approaches, we identified internodes or branches with low degrees of certainty. A combination of strong conflicts in phylogenetic signal and sometimes absence or low informative signals contributed to the conflict in reconstructing the true relationship between the amaranths. We found strong support along the “backbone” relating species in the *Acnida* clade (all nine of the dioecious species except *A. australis* and *A. cannabinus*) and species in the *Amaranthus* clade, and strong support for the sister relationship between both clades, consistent with the nuclear phylogeny in Waselkov et al. [20]. The relationship of *A. australis* + *A. cannabinus* lineage to the other dioecious species however remains obscure, and the topology test of monophyly did not support the placement of both species in the same clade as the other seven dioecious species. Chloroplast genomes are non-recombining and uniparentally inherited, and it is possible that the chloroplast in *A. australis* + *A. cannabinus* lineage was inherited after a hybridization event or chloroplast capture from an ancestor leading to the *Acnida* + *Amaranthus* clades.

Summary coalescent methods are known to be more robust than concatenation methods in the presence of high levels of ILS [60, 61], and we have inferred species tree from the plastid protein-coding genes using a summary coalescent analysis. Genes with short lengths and uninformative loci that is typical of chloroplast genomes may however contribute to gene trees with topology inconsistencies at some branches and a subsequent species tree that is less accurate [62, 63]. Nevertheless, the higher proportion of gene trees (> 50%) concordant with the species tree for Amaranthaceae s.s. (tribes Celosieae, Aerveae, Achyrantheae and Gomphreneae) but not for *Amaranthus* species (Additional file 4: Fig. S4), indicates inherent processes within the *Amaranthus* genus that contribute to conflicting phylogenetic signals. The inclusion of species belonging to these four tribes in our phylogenetic analysis therefore proved informative as it allowed us to validate the relationship of the tribes to Amarantheae. We recovered clades corresponding to relationships between the five tribes previously described in the Angiosperm Phylogeny Group (APG) IV system of classification [64] and previous studies [49, 65, 66].

It is expected that all genes in the plastomes would share the same evolutionary history based on their inheritance patterns. However, recent findings for angiosperms reveal chloroplast genes exhibit well-supported conflict and do not appear to share the same evolutionary history [37, 67]. Plastid gene tree incongruence among five major clades of Amaranthaceae s.l. was recently hypothesized to be likely due to heteroplasmy [49]. It is difficult to determine the exact causes of conflict in plastid gene trees within the *Amaranthus* genus in our study, whether it is a result of varying evolutionary histories of the genes or a result of systematic or other analytical methods e.g., lack of information or misalignment. There is also a debate over the impact of taxon sampling on the accuracy of phylogenetic analysis, with some authors reporting the contribution of low taxon sampling to tree conflicts [68] while others note no impact on tree inference [69] [see Nabhan and Sarkar [70] for a review on taxon sampling controversy]. Nevertheless, we sampled all the species in the dioecious clade (subgenus *Acnida*) as well as several species in the Hybridus clade (subgenus *Amaranthus*) and therefore tree conflicts in our study are not due to low taxon sampling.

Contrary to studies where data partitioning has improved phylogenetic inference [71], topology tests between partitioned and unpartitioned data sets for the 78 CDS revealed no differences between both approaches [72]. However, we recommend data partitioning, as the analysis of the whole plastome data sets yielded branches with high support but also complex conflicts that could not be easily interpreted. While we did not specifically investigate the contribution of tRNA, rRNA and introns by including partitions for them in the phylogenetic tree, the full support for the sister relationship between *A. arenicola* and *A. tuberculatus* using whole plastome alignment, which was not clear from using 78 protein-coding regions, indicates that more signals favoring this relationship could be coming from non-coding regions. Non-coding regions also hold phylogenetic information that could be useful in resolving shallow evolutionary relationships [52, 67]. Their impact on tree inference would need to be further evaluated for the amaranths.

Additional studies into the relationship between the amaranths is required to understand their evolutionary history. Using a *k*-mer-based phylogenomic analysis, Raiyemo et al. [73] reported the relationships among the dioecious *Amaranthus* species. Although, the *k*-mer method was alignment-free and did not model complex evolutionary processes, sister-species relationships (e.g., between *A. australis* and *A. cannabinus*, *A. arenicola* and *A. greggii*, and *A. tuberculatus* and *A. floridanus*) that is congruent with the previous infrageneric classifications based on morphological characteristics were obtained. Nonetheless, phylogenetic studies incorporating morphological data, nuclear genes (perhaps obtained via a hybrid capture-based target enrichment) and mitochondrial data would still be required to enhance our understanding of the evolution of the *Amaranthus* genus and to provide additional insights into tree discordance in the genus [74]. Our work provides a framework for further investigation of the relationship among the amaranths as more species within the genus are sequenced.

### Are *A. palmeri* and *A. watsonii* two species or a single polymorphic species?

Although both *A. palmeri* and *A. watsonii* had long been considered separate species by various authorities [6, 7, 20], the similarity in morphological characteristics, high degree of species range overlap and a low evolutionary distance between both species could indicate a single polymorphic species. Based on Sauer’s [6] reported morphological characteristics, both species are very similar (1 m tall; 5 stamens, 5 tepals, and inner tepal length of 2.5–3 mm for male flowers; 5 tepals with 2–2.5 mm length for female flowers; utricle length of 1.5 mm; 2 or sometimes 3 style branches; and seed with obovate shape and dark reddish brown color), but differ in length of thyrses and shape of leaf blade. Historically, both species were considered important food plant; as a potherb and source of grain for various Indian tribes [6]. Furthermore, Sauer [6] hypothesized that the Colorado River and associated irrigation projects provided the opportunity for *A. watsonii* to mix with *A. palmeri* and move into Southern California as a weed of irrigated fields. Both species are native to California and Arizona and are sympatric in San Bernadino and Imperial counties of California, and Yuma and Maricopa counties of Arizona (https://plants.usda.gov/home) [75].

Stelkens and Seehausen [76] in a study of evolutionary distances for hybridizing species using ITS1 and ITS2 reported a distance of 0.0155 between *A. retroflexus* and *A. cruentus*, which is congruent with the distance values between some closely related monoecious species in our study. The lowest distance in their study was between *Mimulus lewisii* and *M. cardinalis* (0.002), which was much higher than the distance between *A. palmeri* and *A. watsonii* (0.000453) in our study. Although *A. palmeri* is now widespread and has become a troublesome weed of different agricultural systems [14], little is known about *A. watsonii* or interspecific hybridization between both species that may have resulted in novel hybrid traits. Nevertheless, the very low distance between both species in our study based on complete chloroplast genomes and rDNA, in addition to previously reported morphological similarities, indicate that the two species are more genetically related than previously reported. Our study reinforces the taxonomic reconsideration of *A. palmeri* and *A. watsonii* as a single polymorphic species, or perhaps the latter be considered a variety of *A. palmeri*.

## Conclusion

Although, the *Amaranthus* genus has been described as taxonomically challenging to work with due to similarities in species morphology and difficulty in accurate identification, we demonstrate that the use of complementary phylogenetic approaches coupled with proper species identification could be very informative in examining the genus’ complex evolutionary history. We provide additional clarification on the relationships among the dioecious species of the *Amaranthus* genus, which have been conflicting based on previous studies where few molecular markers were used. Important open questions remain for the amaranths: (1) When in the evolutionary and biogeographic time scale did speciation events occurred? (2) When did chloroplast capture events take place? (3) Was there rapid radiation or ancient hybridization in the genus and at what time could this have taken place?

## Methods

### Plant material, DNA extraction and Illumina sequencing

Seeds of seven dioecious species of the *Amaranthus* genus (*A. acanthochiton*, *A. arenicola*, *A. australis, A. cannabinus*, *A. floridanus*, *A. greggii* and *A. watsonii*) were obtained from USDA Germplasm Resources Information Network (GRIN). Voucher specimens of the accessions grown and sequenced have been deposited at the Illinois Natural History Survey (ILLS) Herbarium at the University of Illinois Robert A. Evers Laboratory (Additional file 1: Table S1). The DNA extraction and sequencing procedure have been described previously [73]. Briefly, seeds were grown in containers with a mixture of Sunshine LC1 (Sun Gro Horticulture, 770 Silver Street Agawam, MA) growing mix, soil, peat, and torpedo sand (3:1:1:1 by weight). Two or three young fresh leaves were harvested from each species, flash frozen in liquid nitrogen and stored at – 80 ºC. Genomic DNA was extracted following standard CTAB protocol [77], and DNA integrity was determined using a spectrophotometer (Nanodrop1000 Spectrophotometer, Thermo Fisher Scientific, 81 Wyman Street, Waltham, MA 02451). The DNA samples were submitted to the Roy J. Carver Biotechnology Center at the University of Illinois, Urbana–Champaign for paired-end sequencing (2 × 150 bp) on Illumina NovaSeq6000. Other chloroplast genome assemblies or raw reads of species belonging to the family Amaranthaceae s.s. used in this study were downloaded from the NCBI database and are described further in Additional file 1: Table S2.

### Genome assembly and annotation

Quality of the sequenced raw reads and those from the NCBI database was evaluated with FastQC (https://www.bioinformatics.babraham.ac.uk/projects/fastqc/) and aggregated with MultiQC v1.5 [78]. Low quality bases and adapters were removed with Trimmomatic [79] using parameters: ILLUMINACLIP:TruSeq3-PE.fa:2:30:10:2:True LEADING:3 TRAILING:3 MINLEN:36. The complete chloroplast genomes for the dioecious *Amaranthus* species including other species from the NCBI database were de novo assembled with GetOrganelle v1.7.6.1 [80] using the default parameters, except -R 45. All *Amaranthus* species’ assemblies were seeded with *A. hypochondriacus* reference cp genome (GenBank accession number KX279888). Assembly graphs were visualized with Bandage [81], and synteny plots generated with MUMmer [82] were used to confirm that each assembly had the same SSC orientation as the reference chloroplast genome used to seed the assembly. All assembled chloroplast genomes were then annotated with GeSeq [83]. Annotation steps included the use of the following: BLAT search, ARAGORN v1.2.38, and MPI-MP chloroplast reference set along with the default settings [83]. The annotations were further verified with additional tools, tRNAscan-SE v2.0.7 within GeSeq and a standalone plastid annotation pipeline, Chloe v0.1.0 (https://chloe.plastid.org/annotate.html). Visualization of the chloroplast genome annotation was carried out with the program OGDRAW [84].

### Analysis of simple sequence repeats (SSRs), repetitive sequences and codon usage bias

Microsatellites or simple sequence repeats from the chloroplast genomes were identified with MISA v2.1 (https://webblast.ipk-gatersleben.de/misa/) using the following search parameters: 12, 6, 4, 3, 3, and 3 for mono-, di-, tri-, tetra-, penta-, and hexanucleotide repeats, respectively [85]. Repetitive sequences, including forward, palindromic, reverse, or complementary repeats in the genomes were detected with REPuter (https://bibiserv.cebitec.uni-bielefeld.de/reputer) using a minimal repeat size set to 30 bp and a hamming distance of 3 [86]. Codon usage and relative synonymous codon usage (RSCU) were evaluated with CodonW v1.4.4 [87].

### Comparison of dioecious *Amaranthus* chloroplast genomes

The assembled chloroplast genomes of the nine dioecious *Amaranthus* species were compared to the reference chloroplast genome of *A. hypochondriacus* with mVISTA (https://genome.lbl.gov/vista/mvista/submit.shtml) using the shuffle-LAGAN mode [88]. Comparison of boundaries between the LSC, IR and SSC regions (i.e., LSC/IRb/SSC/IRa) among the chloroplast genomes were carried out with IRSCOPE (https://irscope.shinyapps.io/irapp/) [89]. To avoid data duplication, the IRa region was removed from each of the plastomes prior to alignment. The plastome sequences were then aligned using the FFT-NS-2 method in MAFFT v7.5 [90, 91]. The alignment of the nine dioecious *Amaranthus* species was then used to determine the values of nucleotide variability (π) [92]. Nucleotide variability values were also calculated separately for the alignment of four weedy species (*A. tuberculatus*, *A. palmeri*, *A. hybridus* and *A. retroflexus*). Sliding window analyses were carried out with DnaSp v6.12 [93] using a window length of 800 bp and a step size of 200 bp.

### Phylogenetic analysis

Thirty plastomes belonging to Amaranthaceae s.s., including the newly assembled nine of the dioecious *Amaranthus* species, were used for phylogenetic analyses (Additional file 1: Tables S2, S3). Three species in the family Achatocarpaceae were included as outgroups. Our phylogenetic analyses were focused on understanding the relationship between the dioecious *Amaranthus* species, and therefore did not include other members of the Amaranthaceae s.l.. Phylogenetic analyses were carried out using two datasets: (1) seventy-eight protein-coding sequences (CDS) extracted from the cp assemblies and (2) whole chloroplast genomes with IRa removed. All datasets were aligned with MAFFT v7.5 [90, 91] using the FFT-NS-2 method. The alignments were visually inspected and columns with less than 50% occupancy were removed in Jalview v2.11.2.4 [94]. Alignment statistics were then assessed with MEGA11 [95].

For the concatenated 78 protein-coding sequences, the analyses were carried out with a partitioning scheme–allowing substitution patterns to vary across genes. A Maximum Likelihood (ML) tree implemented in RAxML v8.2.12 [96] was carried out with the alignment using the GTRGAMMA substitution model and 1000 rapid bootstrap replicates. The degree of conflict on each node given the individual gene trees was assessed via the internode certainty all (ICA) which was calculated in RAxML using the extended majority rule consensus tree [97]. In addition, Quartet Sampling [98] with 1000 replicates was carried out to differentiate between strong conflict and weak branch support. The ML bootstrap trees from RAxML were also used to estimate species tree in ASTRAL-III [99].

We complemented our analysis in RAxML by further implementing another ML tree in IQ-TREE v2.1.2 [100], first without partitioning and second with the previous partitioning scheme used, but allowing an optimal model to be determined by ModelFinder [101]. Topology tests between the partitioned and unpartitioned tree was assessed with the approximately unbiased (AU) test [102]. Concordance factors between gene trees and species trees were calculated in IQ-TREE [100]. Additionally, conflicting and concordant bipartitions among gene trees were calculated in Phyparts [103].

Bayesian inference (BI) analyses was carried out with MrBayes v3.2.7 [104] following the partitioning scheme adopted for RAxML. The Markov chain Monte Carlo (MCMC) analyses consisted of two independent runs and four heated chains of 20 million generations each, sampling every 1000 generations using a GTR + G model and a 25% burn-in. The parameters for each partition were unlinked. Convergence of parameter estimates was first assessed by inspecting the average standard deviation of split frequencies in MrBayes, followed by further assessment using Tracer v1.7.2 [105].

For the plastome alignment, ambiguously aligned regions with < 50% occupancy were also inspected and removed from the sequence alignment in Jalview. A ML tree with the optimal model, TVM + F + R2, suggested by ModelFinder was then implemented in IQ-TREE 2 on the alignment without data partitioning. For Bayesian inference phylogeny, the GTR + I + G substitution model was used on the unpartitioned dataset. The Markov chain Monte Carlo (MCMC) analyses consisted of two independent runs and four heated chains of 6 million generations each, sampling every 1000 generations and a 25% burn-in. Parameter convergence was evaluated as previously described. All tree files were visualized and edited in FigTree v1.4.4 (https://github.com/rambaut/figtree) and Dendroscope v3.8.3 [106].

Since bifurcating trees may sometimes be inadequate in depicting the relationships between taxa with reticulation events [107, 108], we further evaluated the relationship among the dioecious *Amaranthus* species with a tree-based bootstrap consensus network that maps bipartition frequencies (e.g., from RAxML bootstrap trees) onto network edges and a distance-based Neighbor-Net algorithm [109] that uses uncorrected *p*-distances in SplitsTree v4.18.3 [110, 111].

We assessed the monophyly of dioecious *Amaranthus* species by constraining all dioecious species to be in one clade following our previous analysis and model in RAxML. Testing the monophyletic dioecious amaranths hypothesis was informed by the observed paraphyly between *A. australis*, *A. cannabinus* and the other seven dioecious species. The per site log-likelihoods of both the unconstrained and constrained trees were computed in RAxML, and used for an approximately unbiased (AU) test in CONSEL v1.20 [112].

### Evolutionary distance between the two dioecious species, *A. palmeri* and *A. watsonii*

*Amaranthus palmeri* and *A. watsonii* are two dioecious species with very similar morphological characteristics and exhibited sister relationships in previous phylogenies [20]. To understand the relationship between both species, we used the whole plastome alignment (minus IRa) as input for MEGA11 [95] to calculate evolutionary distances (uncorrected *p*-distances). Additionally, we assembled the nuclear ribosomal DNA (rDNA) genes, 18S (small subunit, SSU), 5.8S, 26S (large subunit, LSU) and their internal transcribed spacers, ITS1 and ITS2 from short reads sequences of the dioecious species with GetOrganelle v1.7.6.1 [80]. Each of the rDNA genes were identified from the assembly using Rfam 14.8 [[113, 114]; http://rfam.xfam.org/] and the ITS regions were further verified with the tool, ITSx [115]. Both the complete ITS region (18S-ITS1-5.8S-ITS2-26S) and the full rDNA were then aligned using MAFFT. To reduce assembly artifacts due to the difficulty in assembling externally transcribed spacer (ETS) and intergenic spacer (IGS) from short reads, we removed columns with < 50% occupancy from the full rDNA alignment. Evolutionary distances were then calculated as previously described.

## Supplementary Information

**Additional file 1: Table S1.** Sequence information for dioecious *Amaranthus* species used in plastome assembly. **Table S2.** Sequence information for species used in phylogenomic analysis. **Table S3.** Chloroplast genome features of additional species assembled in this study. **Table S4.** Assembly size of nuclear rDNA region of species assembled in this study.

**Additional file 2: Table S5.** Relative synonymous codon usage of 78 protein-coding genes in the chloroplast genome of *Amaranthus tuberculatus*.

**Additional file 3: Figure S1.** Sliding window analysis of nucleotide diversity among nineteen chloroplast genomes of *Amaranthus* species.

**Additional file 4: Figure S2.** Phylogenetic tree of *Amaranthus* species and other species in Amaranthaceae s.s. from RAxML based on 78 plastid protein-coding genes. **Figure S3.** Phylogenetic tree of *Amaranthus* species and other species in Amaranthaceae s.s. from IQ-TREE based on 78 plastid protein-coding genes. **Figure S4.** Phylogenetic tree of *Amaranthus* species based on maximum likelihood analysis of 78 plastid protein-coding genes in IQ-TREE. **Figure S5.** Bootstrap consensus network inferred from the maximum likelihood tree analysis for *Amaranthus* species and other species in Amaranthaceae s.s. based on whole chloroplast genomes. **Figure S6.** NeighborNet splits graph of *Amaranthus* species and other species in Amaranthaceae s.s. based on whole chloroplast genomes.

**Additional file 5.** Estimates of evolutionary divergence between ITS sequences of 14 species.

**Additional file 6.** Estimates of evolutionary divergence between nuclear rDNA sequence assembly of 14 *Amaranthus* species.

## Data Availability

Raw reads data generated or analyzed in this study are available through the National Center for Biotechnology Information (NCBI) under project number PRJNA836903. Assembled complete chloroplast genomes and alignments are available on figshare (https://doi.org/10.6084/m9.figshare.21936021). Voucher specimens of the accessions grown and sequenced have been deposited at the Illinois Natural History Survey (ILLS) Herbarium at the University of Illinois Robert A. Evers Laboratory (Additional file 1: Table S1).
