# Supplementary material for: Comparative analysis of dioecious Amaranthus plastomes and phylogenomic implications within Amaranthaceae s.s
Source: BMC Ecol Evol. 2023 May 6;23:15. doi: 10.1186/s12862-023-02121-1 (PMC10164334; doi:10.1186/s12862-023-02121-1)
Supplement: Supplementary file 1 — Additional file 1: Table S1. Sequence information for dioecious Amaranthus species used in plastome assembly. Table S2. Sequence information for species used in phylogenomic analysis. Table S3. Chloroplast genome features of additional species assembled in this study. Table S4. Assembly size of nuclear rDNA region of species assembled in this study. [file 12862_2023_2121_MOESM1_ESM.docx]

**Table S1.** Sequence information for dioecious *Amaranthus* species used in plastome assembly.

| Species | Accession ID | SRA number | Voucher specimen number | Sequencing platform |
| --- | --- | --- | --- | --- |
| *Amaranthus acanthochiton* | PI 632239 | SRR19158647 | ILLS00148924 | Hiseq 6000 |
| *Amaranthus arenicola* | PI 599670 | SRR19158645 | ILLS00148920 | Hiseq 6000 |
| *Amaranthus floridanus* | PI 553078 | SRR19158641 | ILLS00148930 | Hiseq 6000 |
| *Amaranthus greggii* | PI 667170 | SRR19158639 | ILLS00148922 | Hiseq 6000 |
| *Amaranthus australis* | PI 553077 | SRR19158644 | ILLS00148921 | Hiseq 6000 |
| *Amaranthus cannabinus* | PI 641041 | SRR19158642 | ILLS00148926 | Hiseq 6000 |
| *Amaranthus watsonii* | PI 633593 | SRR19158646 | ILLS00148929 | Hiseq 6000 |

**Table S2.** Sequence information for species used in phylogenomic analysis.

| Species | Cp genome available | Accession number | SRA number | Sequencing platform | Cp genome assembled |
| --- | --- | --- | --- | --- | --- |
| *Alternanthera philoxeroides* | Yes | MK795965 |  |  |  |
| *Alternanthera nodiflora* | Yes | MK410015 |  |  |  |
| *Achyranthes bidentata* | Yes | MN652923 |  |  |  |
| *Achyranthes longifolia* | Yes | MN953049 |  |  |  |
| *Celosia cristata* | Yes | MK470118 |  |  |  |
| *Celosia argentea* | Yes | MK397861 |  |  |  |
| *Deeringia amaranthoides* | Yes | MK397865 |  |  |  |
| *Ptilotus latifolius* | Yes | MK410027 |  |  |  |
| *Ptilotus villosiflorus* | Yes | MK410026 |  |  |  |
| *Ptilotus chamaecladus* | Yes | MK410025 |  |  |  |
| *Aerva javanica* | Yes | MK410028 |  |  |  |
| *Amaranthus tuberculatus* |  |  | SRR12075660 | Hiseq 3000 | Yes |
| *Amaranthus palmeri* |  |  | SRR5012826 | Hiseq 2500 | Yes |
| *Amaranthus retroflexus* | Yes | MW646089 |  |  |  |
| *Amaranthus cruentus* | Yes | MG836507 |  |  |  |
| *Amaranthus caudatus* | Yes | MG836508 |  |  |  |
| *Amaranthus dubius* | Yes | MZ397802 |  |  |  |
| *Amaranthus hybridus* |  |  | SRR12075659 | Hiseq 3000 | Yes |
| *Amaranthus quitensis* |  |  | ERR3021376 | Hiseq 4000 | Yes |
| *Amaranthus hypochondriacus* | Yes | KX279888 |  |  |  |
| *Amaranthus tricolor* | Yes | KX094399 |  |  |  |
| *Amaranthus viridis* | Yes | MW679034 |  |  |  |
| *Amaranthus polygonoides* | Yes | MT472619 |  |  |  |
| *Achatocarpus pubescens* | Yes | MK397909 |  |  |  |
| *Achatocarpus nigricans* | Yes | MK397908 |  |  |  |
| *Phaulothamnus spinescens* | Yes | MH286322 |  |  |  |

**Table S3.** Chloroplast genome features of additional species assembled in this study.

| Species | Length (bp) | Coverage depth (x) | LSC (bp) | SSC (bp) | IR (bp) | GC (%) | Number of genes | | | |
| --- | --- | --- | --- | --- | --- | --- | --- | --- | --- | --- |
|  |  |  |  |  |  |  | **Protein-coding** | **tRNA** | **rRNA** | **Total** |
| *Amaranthus quitensis* | 150,691 | 413.8 | 84,063 | 17,948 | 24,340 | 36.58 | 78 | 30 | 4 | 112 |
| *Amaranthus hybridus* | 150,798 | 519.5 | 84,107 | 17,989 | 24,351 | 36.56 | 78 | 30 | 4 | 112 |

**Table S4.** Assembly size of nuclear rDNA region of species assembled in this study.

| Species | Assembly size (bp) |
| --- | --- |
| *Amaranthus acanthochiton* | 11,296 |
| *Amaranthus arenicola* | 10,399 |
| *Amaranthus australis* | 11,365 |
| *Amaranthus cannabinus* | 11,128 |
| *Amaranthus floridanus* | 11,582 |
| *Amaranthus tuberculatus* | 10,911 |
| *Amaranthus greggii* | 11,541 |
| *Amaranthus watsonii* | 9,894 |
| *Amaranthus palmeri* | 9,909 |
| *Amaranthus hybridus* | 10,446 |
| *Amaranthus caudatus* | 11,403 |
| *Amaranthus quitensis* | 10,409 |
| *Amaranthus hypochondriacus* | 10,965 |
| *Amaranthus cruentus* | 10,279 |
