# Supplementary material for: Comparative analysis of dioecious Amaranthus plastomes and phylogenomic implications within Amaranthaceae s.s
Source: BMC Ecol Evol. 2023 May 6;23:15. doi: 10.1186/s12862-023-02121-1 (PMC10164334; doi:10.1186/s12862-023-02121-1)
Supplement: Supplementary file 2 — Additional file 2: Table S5. Relative synonymous codon usage of 78 protein-coding genes in the chloroplast genome of Amaranthus tuberculatus. [file 12862_2023_2121_MOESM2_ESM.docx]

**Table S5.** Relative synonymous codon usage (RSCU) of 78 protein-coding genes in the chloroplast genome of *Amaranthus tuberculatus*.

| Amino acid | Codon | Count | RSCU | Amino acid | Codon | Count | RSCU |
| --- | --- | --- | --- | --- | --- | --- | --- |
| Phe | UUU | 962 | 1.28 | Tyr | UAU | 719 | 1.31 |
| Phe | UUC | 547 | 0.72 | Tyr | UAC | 380 | 0.69 |
| Leu | UUA | 584 | 1.57 | * | UAA | 455 | 1.17 |
| Leu | UUG | 503 | 1.35 | * | UAG | 302 | 0.77 |
| Leu | CUU | 462 | 1.24 | His | CAU | 291 | 1.33 |
| Leu | CUC | 210 | 0.56 | His | CAC | 147 | 0.67 |
| Leu | CUA | 299 | 0.80 | Gln | CAA | 504 | 1.43 |
| Leu | CUG | 175 | 0.47 | Gln | CAG | 199 | 0.57 |
| Ile | AUU | 825 | 1.46 | Asn | AAU | 770 | 1.38 |
| Ile | AUC | 371 | 0.66 | Asn | AAC | 344 | 0.62 |
| Ile | AUA | 498 | 0.88 | Lys | AAA | 881 | 1.37 |
| Met | AUG | 421 | 1.00 | Lys | AAG | 405 | 0.63 |
| Val | GUU | 365 | 1.45 | Asp | GAU | 492 | 1.46 |
| Val | GUC | 162 | 0.64 | Asp | GAC | 183 | 0.54 |
| Val | GUA | 311 | 1.24 | Glu | GAA | 641 | 1.44 |
| Val | GUG | 167 | 0.66 | Glu | GAG | 249 | 0.56 |
| Ser | UCU | 406 | 1.19 | Cys | UGU | 370 | 1.11 |
| Ser | UCC | 328 | 0.96 | Cys | UGC | 295 | 0.89 |
| Ser | UCA | 478 | 1.40 | * | UGA | 414 | 1.06 |
| Ser | UCG | 259 | 0.76 | Trp | UGG | 507 | 1.00 |
| Pro | CCU | 206 | 1.06 | Arg | CGU | 170 | 0.70 |
| Pro | CCC | 164 | 0.84 | Arg | CGC | 90 | 0.37 |
| Pro | CCA | 234 | 1.20 | Arg | CGA | 255 | 1.05 |
| Pro | CCG | 173 | 0.89 | Arg | CGG | 172 | 0.71 |
| Thr | ACU | 284 | 1.15 | Ser | AGU | 314 | 0.92 |
| Thr | ACC | 200 | 0.81 | Ser | AGC | 269 | 0.79 |
| Thr | ACA | 336 | 1.36 | Arg | AGA | 460 | 1.89 |
| Thr | ACG | 169 | 0.68 | Arg | AGG | 313 | 1.29 |
| Ala | GCU | 204 | 1.31 | Gly | GGU | 256 | 0.91 |
| Ala | GCC | 127 | 0.82 | Gly | GGC | 204 | 0.73 |
| Ala | GCA | 186 | 1.20 | Gly | GGA | 352 | 1.26 |
| Ala | GCG | 104 | 0.67 | Gly | GGG | 308 | 1.10 |

*Amino acids in asterisk represents termination codons.
