# Supplementary material for: Comparative analysis of dioecious Amaranthus plastomes and phylogenomic implications within Amaranthaceae s.s
Source: BMC Ecol Evol. 2023 May 6;23:15. doi: 10.1186/s12862-023-02121-1 (PMC10164334; doi:10.1186/s12862-023-02121-1)
Supplement: Supplementary file 3 — Additional file 3: Figure S1. Sliding window analysis of nucleotide diversity among nineteen chloroplast genomes of Amaranthus species. [file 12862_2023_2121_MOESM3_ESM.docx]

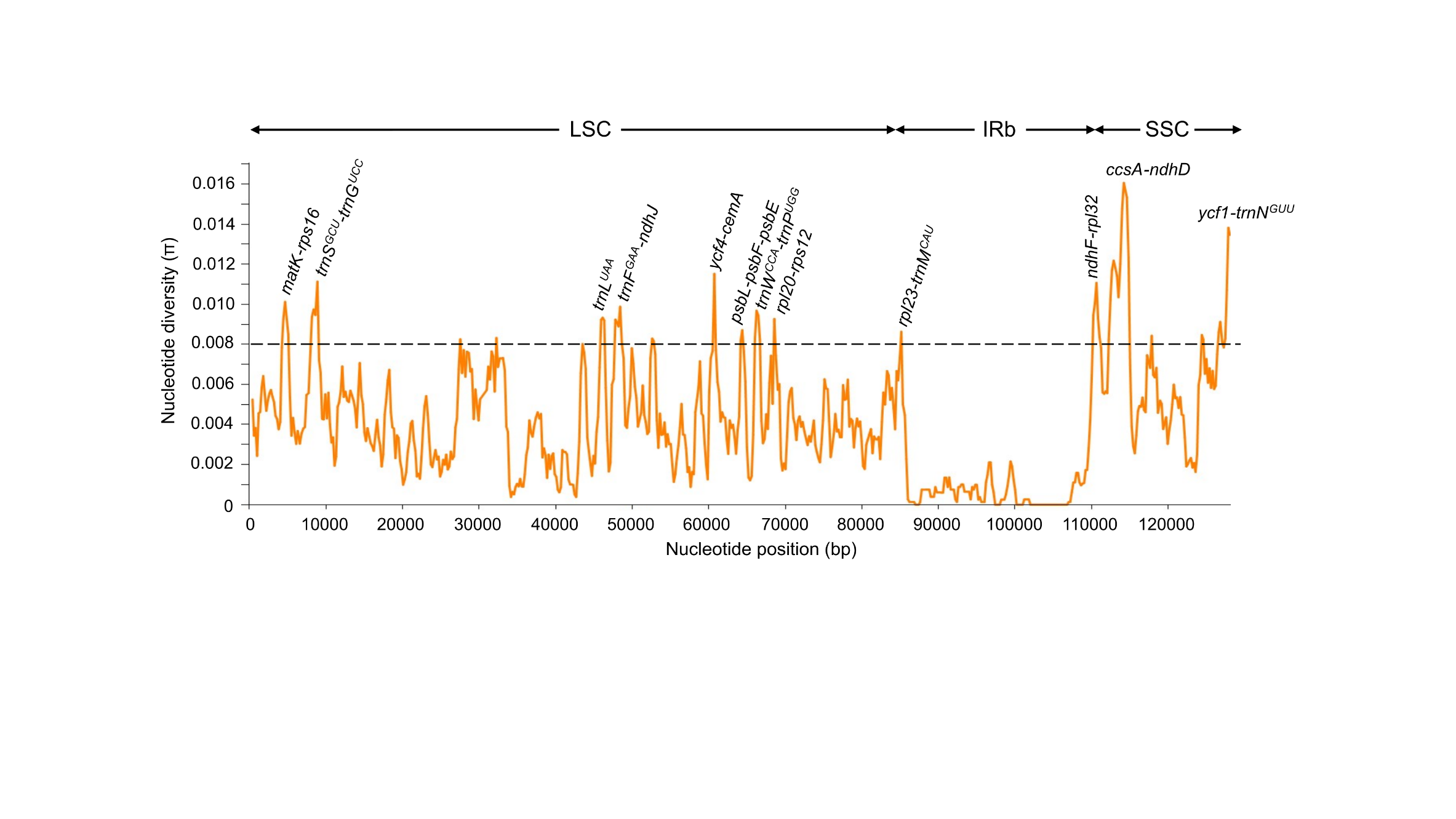


**Figure S1.** Sliding window analysis of nucleotide diversity among nineteen chloroplast genomes of *Amaranthus* species. Eleven *Amaranthus* cp genomes were assembled in this study while eight genomes were obtained from NCBI database. Window length: 800 bp; step size: 200 bp.
