# Supplementary material for: Comparative analysis of dioecious Amaranthus plastomes and phylogenomic implications within Amaranthaceae s.s
Source: BMC Ecol Evol. 2023 May 6;23:15. doi: 10.1186/s12862-023-02121-1 (PMC10164334; doi:10.1186/s12862-023-02121-1)
Supplement: Supplementary file 4 — Additional file 4: Figure S2. Phylogenetic tree of Amaranthus species and other species in Amaranthaceae s.s. from RAxML based on 78 plastid protein-coding genes. Figure S3. Phylogenetic tree of Amaranthus species and other species in Amaranthaceae s.s. from IQ-TREE based on 78 plastid protein-coding genes. Figure S4. Phylogenetic tree of Amaranthus species based on maximum likelihood analysis of 78 plastid protein-coding genes in IQ-TREE. Figure S5. Bootstrap consensus network inferred from the maximum likelihood tree analysis for Amaranthus species and other species in Amaranthaceae s.s. based on whole chloroplast genomes. Figure S6. NeighborNet splits graph of Amaranthus species and other species in Amaranthaceae s.s. based on whole chloroplast genomes. [file 12862_2023_2121_MOESM4_ESM.docx]

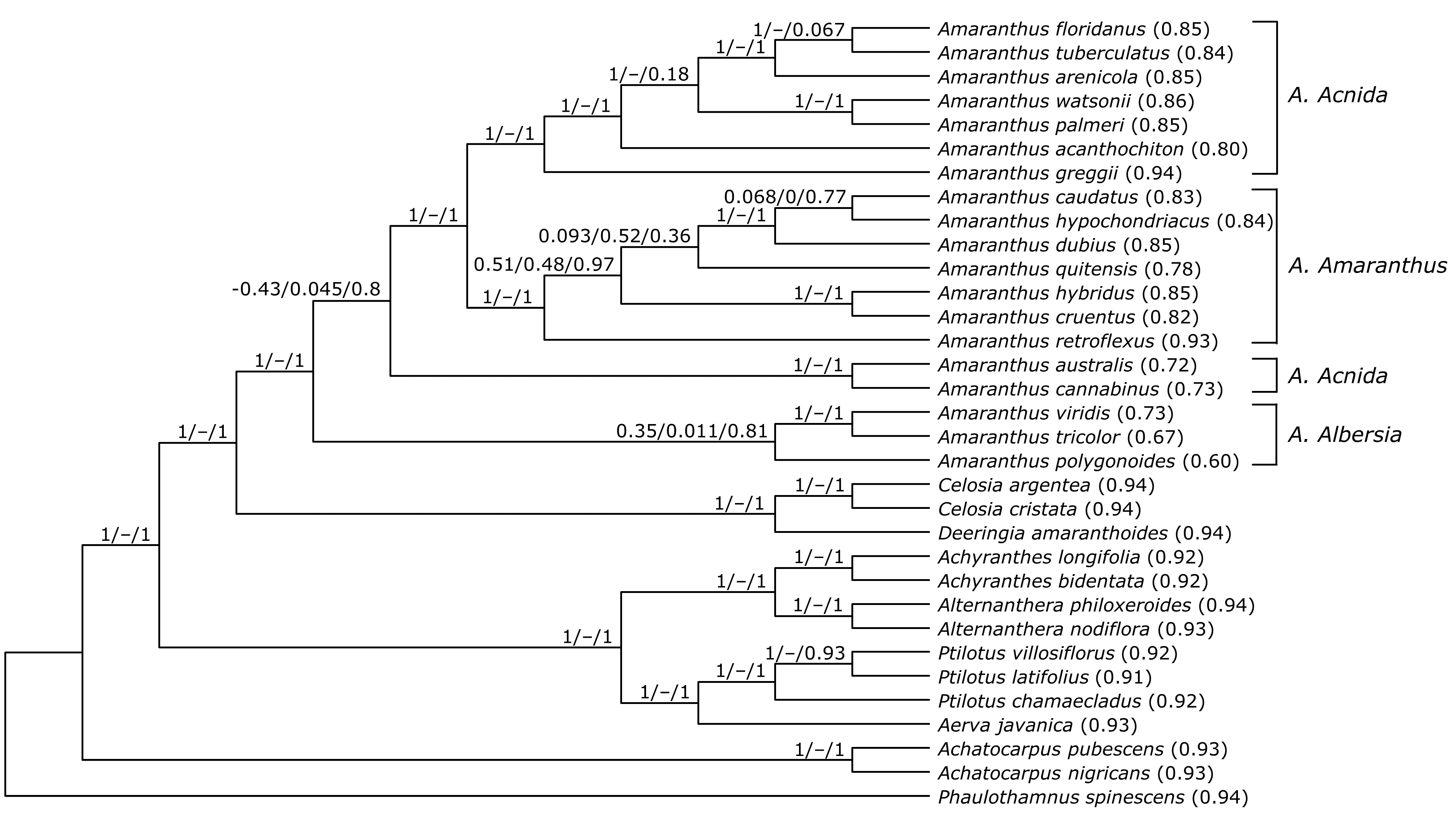


**Figure S2.** Phylogenetic tree of *Amaranthus* species and other species in Amaranthaceae s.s. from RAxML based on 78 plastid protein-coding genes. Numbers above branches represent Quartet sampling internal node score (Quartet Concordance/Quartet Differential/Quartet Informativeness). Values in parentheses after taxa represent Quartet Fidelity (QF) score.


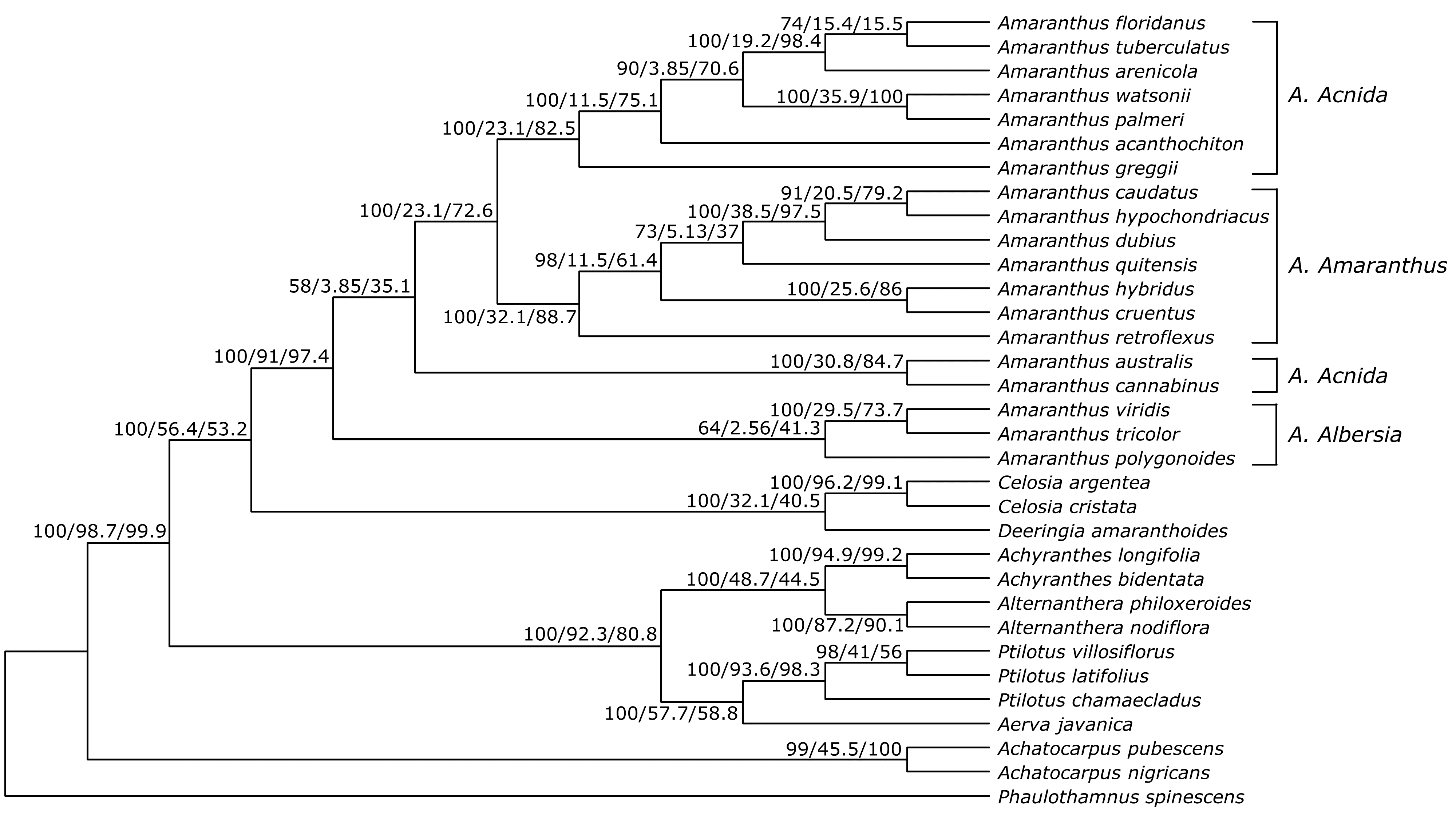


**Figure S3.** Phylogenetic tree of *Amaranthus* species and other species in Amaranthaceae s.s. from IQ-TREE based on 78 plastid protein-coding genes. Numbers above branches represent concordance factors (Bootstrap support value/gene concordance factor gCF/site concordance factor sCF).


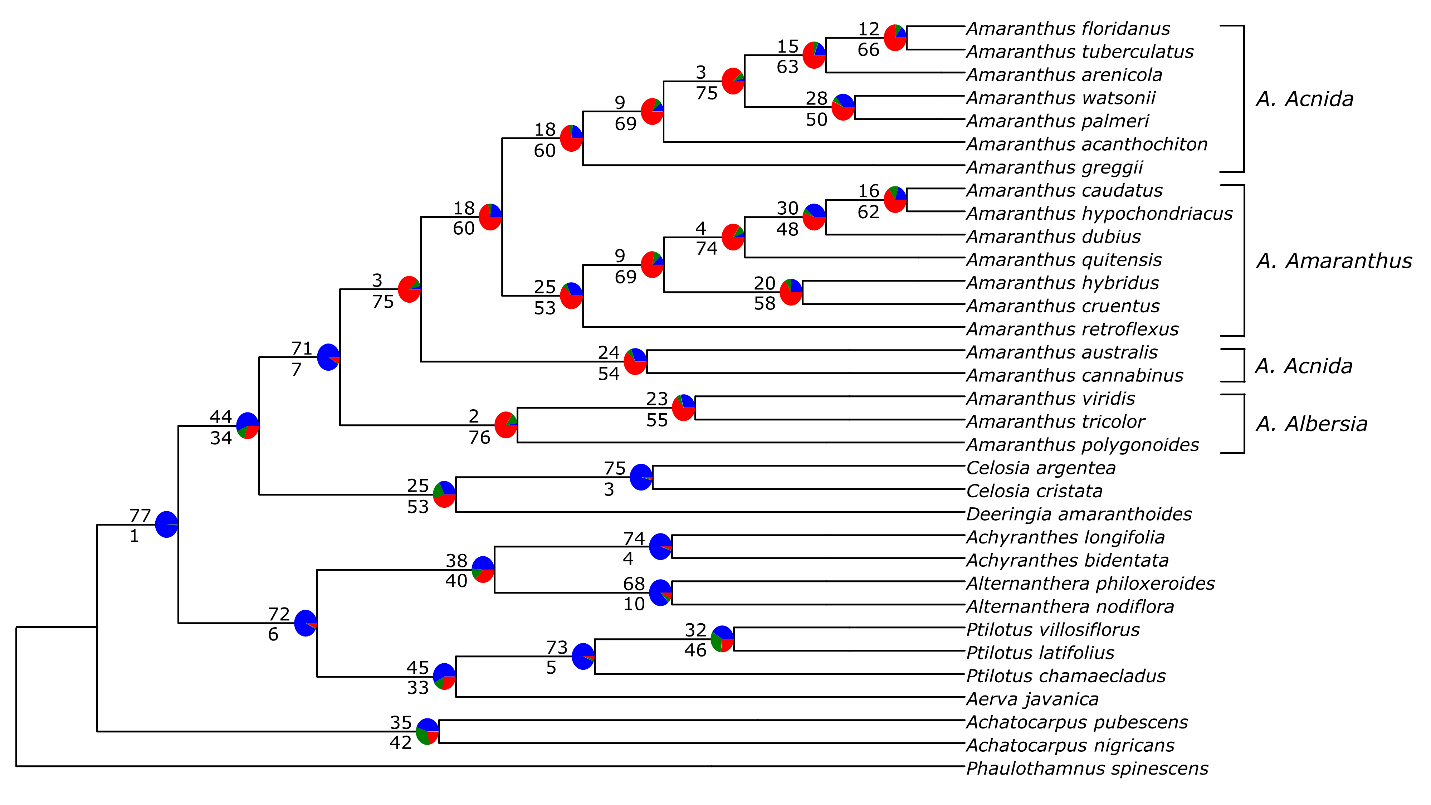


**Figure S4.** Cladogram of *Amaranthus* species based on maximum likelihood analysis of 78 plastid protein-coding genes in IQ-TREE. Numbers above branches represent the number of gene trees that are concordant with the species tree at each node, while numbers below branches represent the number of gene trees that are conflicting with the species tree at each node. Pie charts at each node represent the proportion of gene trees that support that clade (blue), the proportion that support the main alternative for that clade (green) and the proportion that support the remaining alternatives (red).


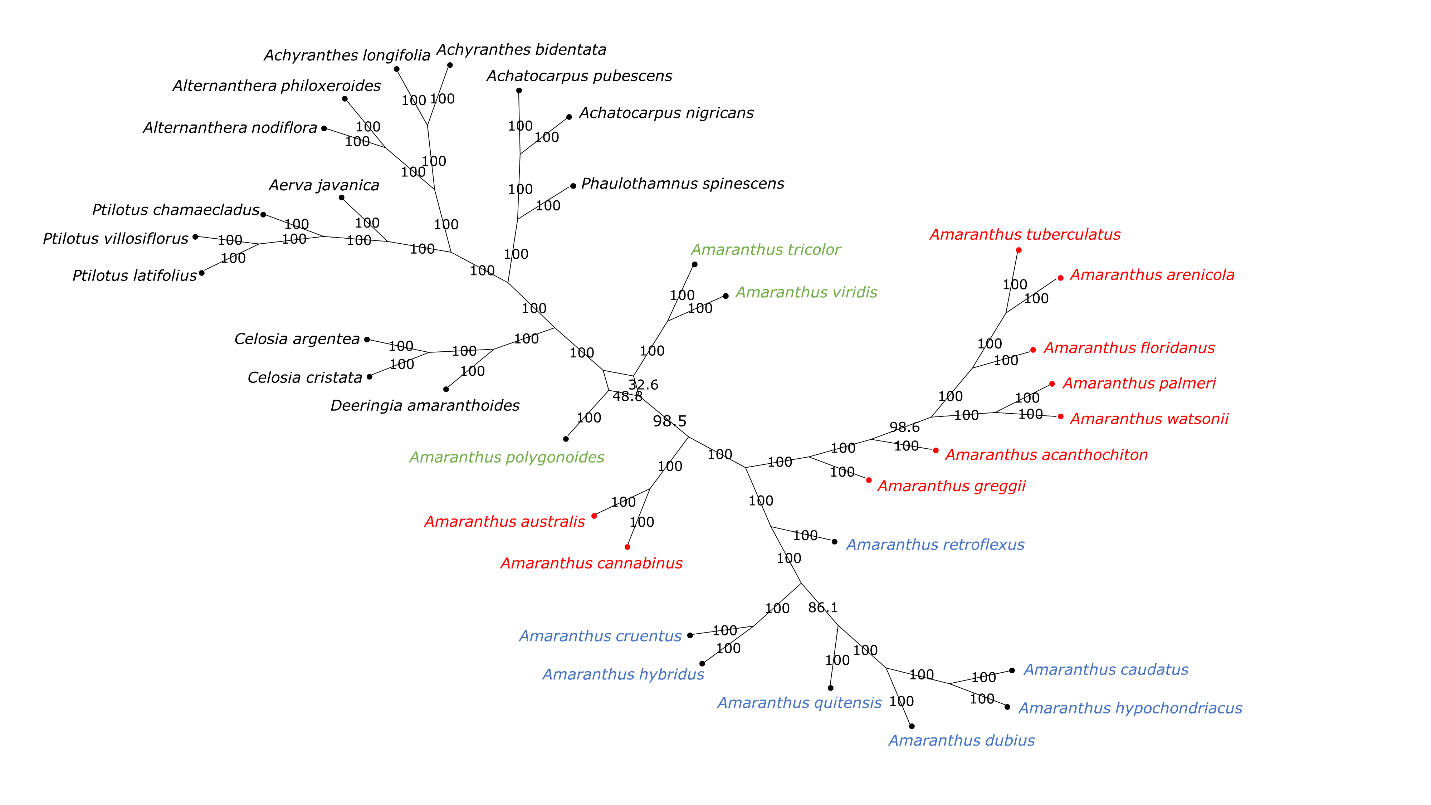


**Figure S5.** Bootstrap consensus network inferred from the maximum likelihood tree analysis for *Amaranthus* species and other species in Amaranthaceae s.s. based on whole chloroplast genomes. Filtering threshold was 0.2 i.e., display splits or taxon bipartitions that occurred in at least 20% of the bootstrap replicates. Numbers on edges of the splits network are bootstrap support values. Species in red denotes subgenus *Acnida* while terminal tips in red are species with chloroplast genome assembled in this study. Species in blue represents the subgenus *Amaranthus* while species in green represent subgenus *Albersia*.


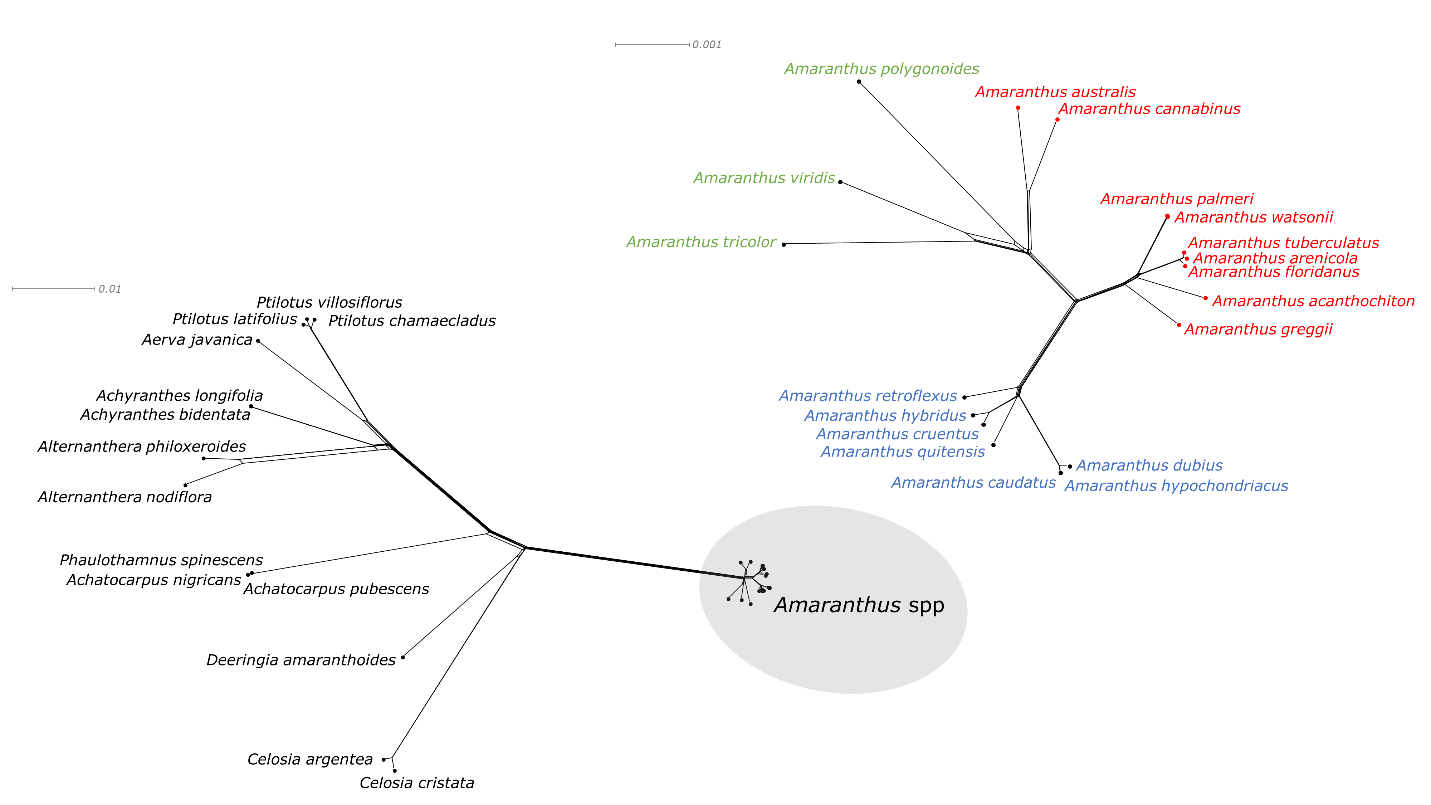


**Figure S6.** NeighborNet splits graph of *Amaranthus* species and other species in Amaranthaceae s.s. based on whole chloroplast genomes. Splits graph of *Amaranthus* spp in the gray circle is enlarged in the top-right corner. Species in red denotes subgenus *Acnida* while terminal tips in red are species with chloroplast genome assembled in this study. Species in blue represents the subgenus *Amaranthus* while species in green represent subgenus *Albersia*. Scale bars (substitutions per site) are presented at the top-left corner of the graphs.
